# Supplementary material for: A Metzincin and TIMP-Like Protein Pair of a Phage Origin Sensitize Listeria monocytogenes to Phage Lysins and Other Cell Wall Targeting Agents
Source: Microorganisms. 2021 Jun 18;9(6):1323. doi: 10.3390/microorganisms9061323 (PMC8235301; doi:10.3390/microorganisms9061323)
Supplement: Supplementary file 1 [file microorganisms-09-01323-s001.zip › microorganisms-1231178-supplementary.pdf]

Supplementary Figure S1

A.

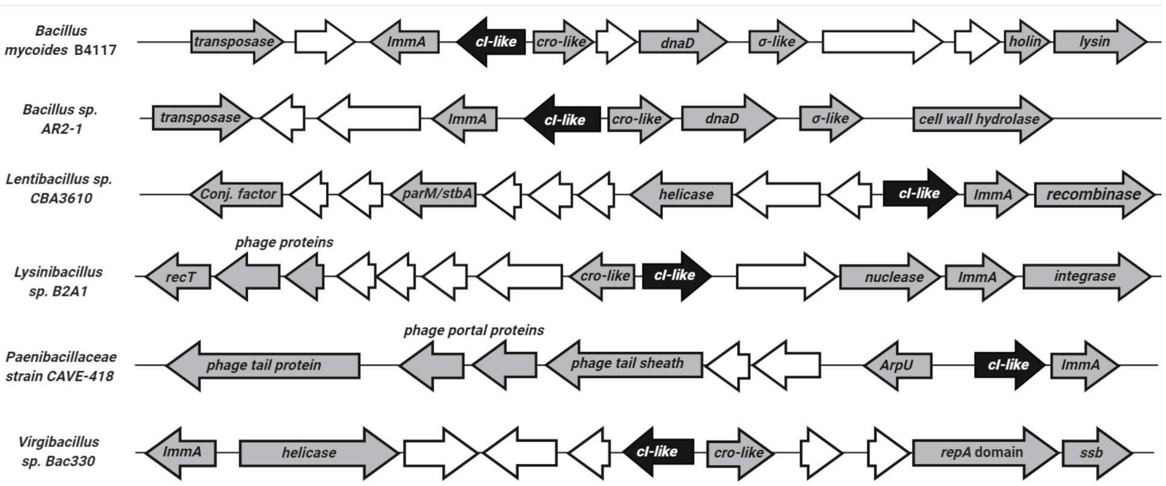

B.

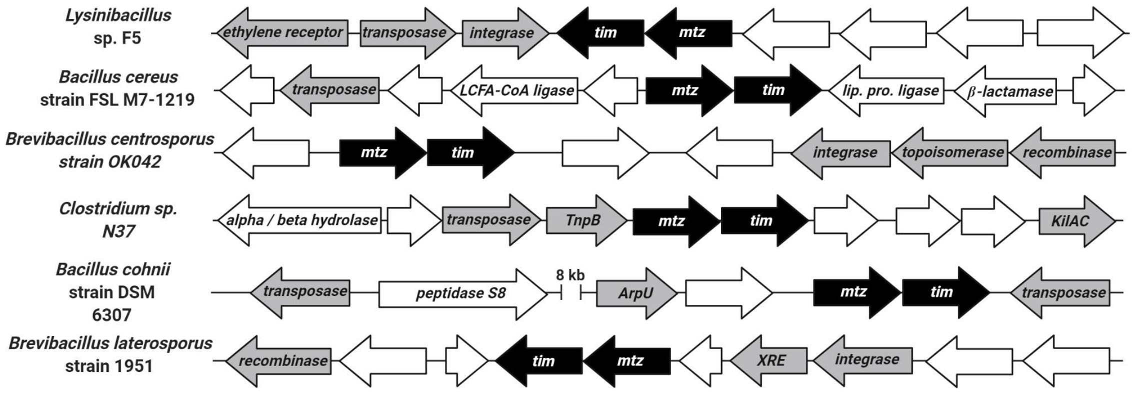

Figure S1. (A) **Bioinformatic analysis of *cI*-like homologues in Gram-positive bacteria (Firmicutes).** Schematic representation of the *cI-like* homologues (~ 60% similarity, not including the HTH domain, belonging to the HipB superfamily) and proximal genes in Firmicutes bacteria related to *Lm*. The *cI-like* homologues are found proximal to mobile genetic element-associated genes and *immA* antirepressors. Black arrows represent the *cI-like* homologues, gray arrows represent mobile genetic element genes. (B) In Firmicutes bacteria related to *Listeria,* gene pairs highly similar to the metzincin-TIMP-like pair are found proximal to mobile genetic element genes. Black arrows represent the *cro*-*cI*-like/metzincin-TIMP-like genes, gray arrows represent mobile genetic element genes.

Supplementary Figure S2

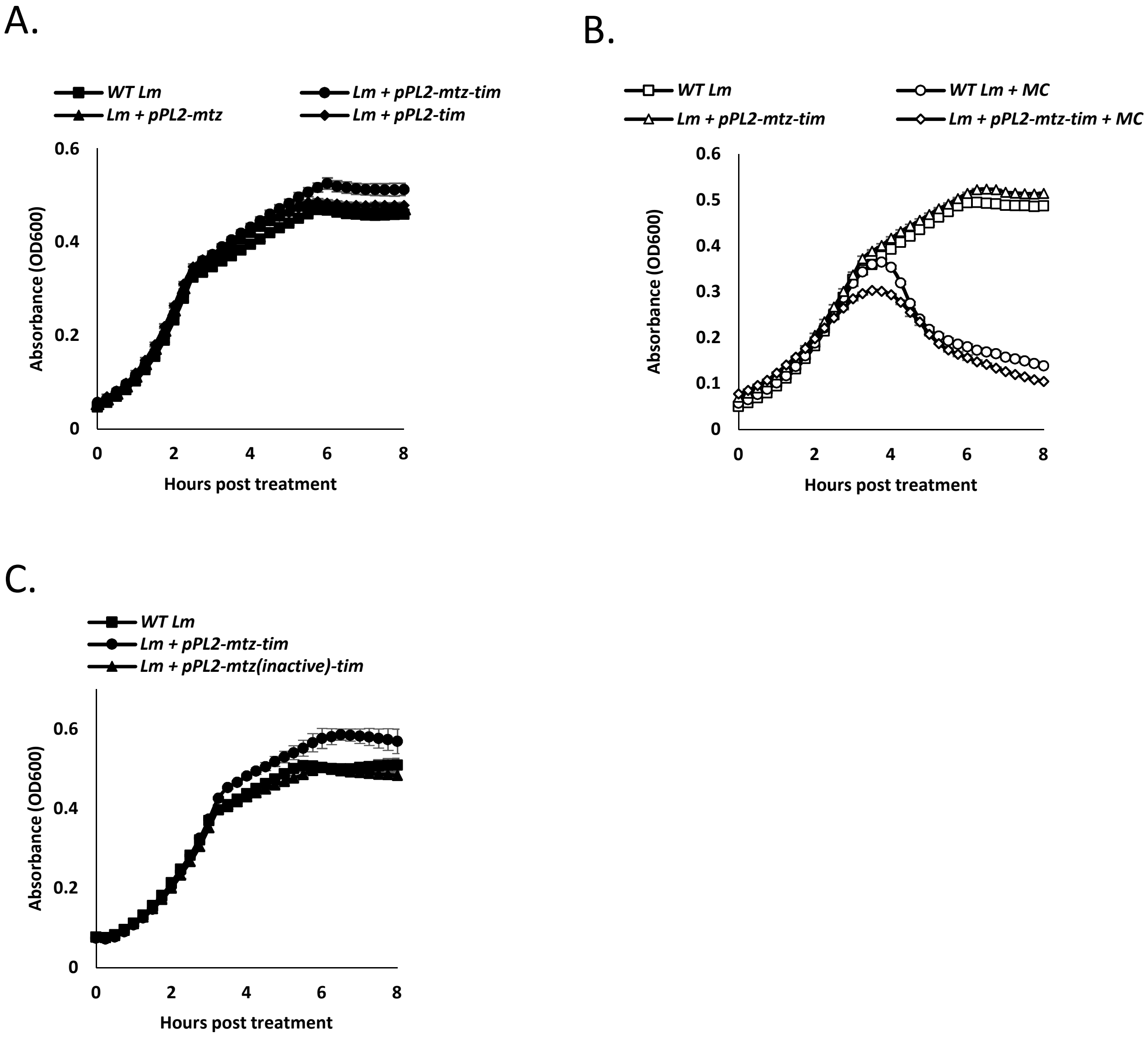

Figure S2. Ectopic expression of Mtz-Tim does not affect *Lm* growth in BHI at 37°C. (A) Growth analysis in BHI media of WT *Lm*, and WT *Lm* harboring pPL2 integrative plasmid expressing *mtz, tim* or both from an inducible p_{tetR} promoter (pPL2-*mtz,* pPL2-*tim*, and pPL2-*mtz-tim* respectively) at 37°C. (B) Growth analysis in BHI media of WT *Lm*, and WT *Lm* harboring pPL2 integrative plasmid expressing *mtz* and *tim* from an inducible p_{tetR} promoter (pPL2-*mtz-tim*) with and without MC treatment at 30°C. (C) Growth analysis in BHI media of WT *Lm*, and WT *Lm* harboring pPL2 integrative plasmid expressing *mtz-tim* or *mtz* mutated at position E136A with *tim* from an inducible p_{tetR} promoter (pPL2-*mtz-tim* and pPL2-*mtz(inactive)-tim*, respectively) at 37°C. Data in A-C represent three independent experiments and error bars represent the standard deviation of triplicates.

Supplementary Figure S3

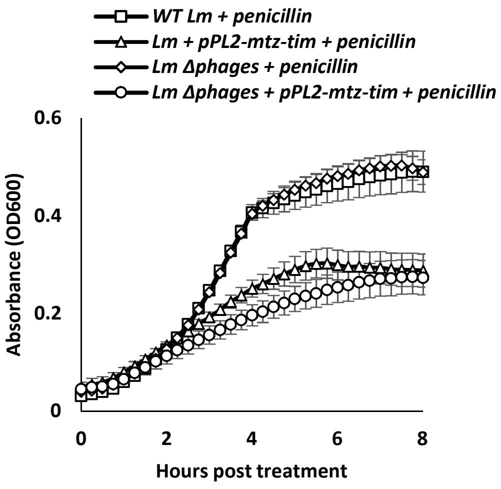

Figure S3. The sensitivity of Mtz-Tim expressing bacteria to penicillin is independent of the phage elements. Growth analysis in BHI media of *Lm* strain deleted of the phage elements (*Δphages*) and WT *Lm* harboring pPL2 plasmid expressing *mtz-tim* from an inducible p_{tetR} promoter (pPL2-*mtz-tim*) at 37°C, with and without addition of penicillin. Data represent three independent experiments and error bars represent the standard deviation of triplicates.

Supplementary Figure S4

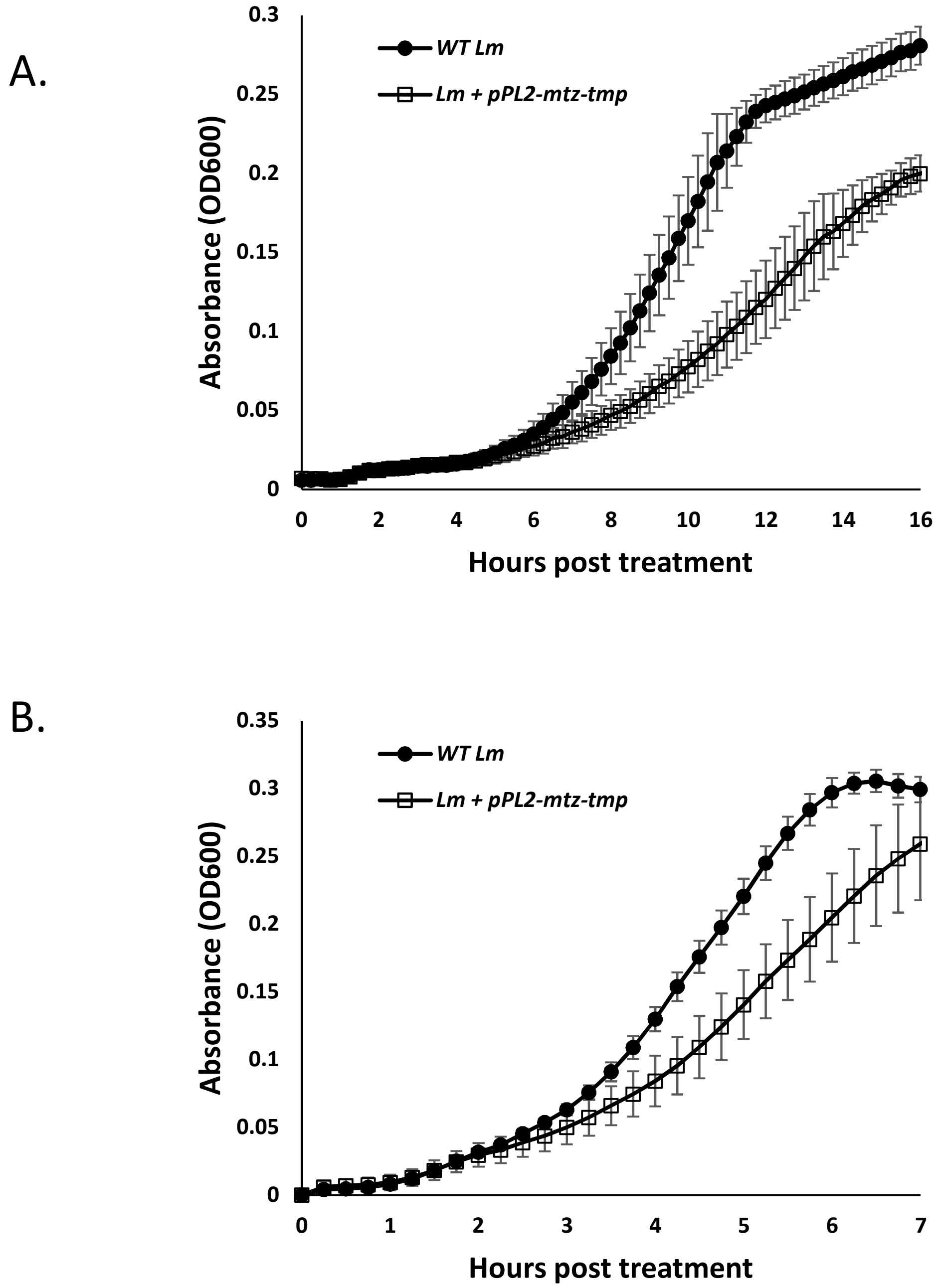

Figure S4. Ectopic expression of Mtz-Tim sensitizes *Lm* to cell-wall acting antibiotics. Growth analysis in BHI media of WT *Lm* and WT *Lm* harboring pPL2 plasmid expressing *mtz-tim* from an inducible p_{tetR} promoter (pPL2-*mtz-tim*) at 37°C, with and without addition of 250 µg/ml of bacitracin (A) and 12 µg/ml D-cycloserine (B). Data represent an average of three independent experiments and error bars represent the standard deviation.

Supplementary Figure S5

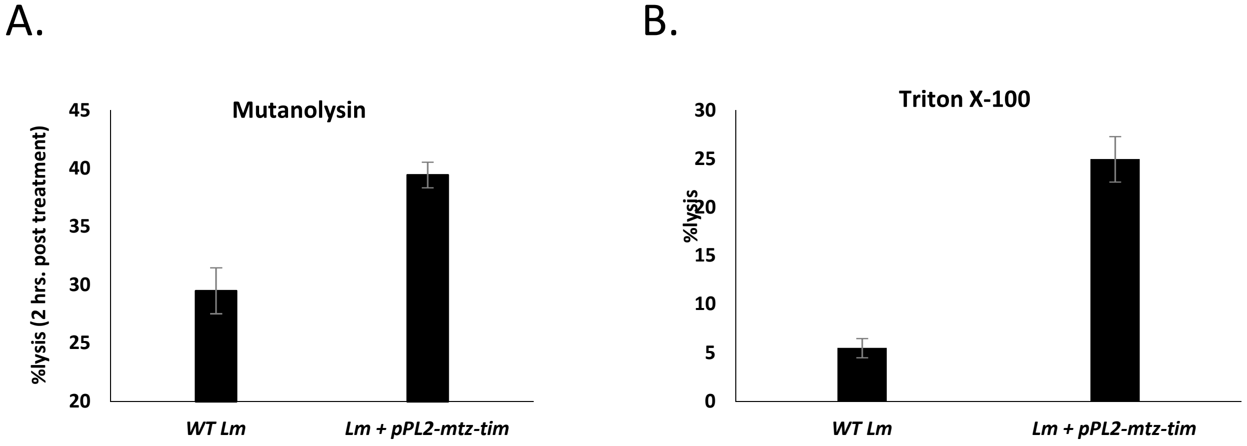

Figure S5. Ectopic expression of Mtz-Tim sensitizes *Lm* to lysis caused by mutanolysin or Triton X-100. Growth analysis in BHI media at 37°C of WT *Lm*, and WT *Lm* harboring pPL2 plasmid expressing *mtz-tim* from an inducible p_{tetR} promoter (pPL2-*mtz-tim*) with and without addition of mutanolysin (A) or Triton X-100 (B). Data is presented as percentage of lysis at 2 hours after treatment. The data represent three independent experiments, error bars represent the standard deviation of triplicates.
